# The delay-reward heuristic: What do people expect in intertemporal choice tasks?

**Published:** 2020-09

**Authors:** William J. Skylark, Kieran T.F. Chan, George D. Farmer, Kai W. Gaskin, Amelia R. Miller

**Affiliations:** * Department of Psychology, University of Cambridge; † Division of Neuroscience & Experimental Psychology, University of Manchester

**Keywords:** delay discounting, risk-reward heuristic, delay-reward heuristic, decision-making, intertemporal choice

## Abstract

Recent research has shown that risk and reward are positively correlated in many environments, and that people have internalized this association as a “risk-reward heuristic”: when making choices based on incomplete information, people infer probabilities from payoffs and vice-versa, and these inferences shape their decisions. We extend this work by examining people’s expectations about another fundamental trade-off—that between monetary reward and delay. In 2 experiments (total N = 670), we adapted a paradigm previously used to demonstrate the risk-reward heuristic. We presented participants with intertemporal choice tasks in which either the delayed reward or the length of the delay was obscured. Participants inferred larger rewards for longer stated delays, and longer delays for larger stated rewards; these inferences also predicted people’s willingness to take the delayed option. In exploratory analyses, we found that older participants inferred longer delays and smaller rewards than did younger ones. All of these results replicated in 2 large-scale pre-registered studies with participants from a different population (total N = 2138). Our results suggest that people expect intertemporal choice tasks to offer a trade-off between delay and reward, and differ in their expectations about this trade-off. This “delay-reward heuristic” offers a new perspective on existing models of intertemporal choice and provides new insights into unexplained and systematic individual differences in the willingness to delay gratification.

## 1 Introduction

Would you rather receive $10 now or $15 in 3 months’ time? Thousands of studies have used this kind of question to investigate the processes by which humans choose between outcomes that differ in when they will occur. The central finding is that, for positive outcomes, deferment renders a positive outcome less attractive – that is, delays lead to discounting of the reward. To avoid dynamic inconsistency (changes in preference between the sooner and later options as they both draw nearer to the present moment), discounting should follow an exponential function: *F* = *Ae* ^–*kt*^, where *F* is the subjective value now of an amount *A* that occurs at time *t* from now, and *k* is a parameter that determines the steepness of the discounting (Samuelson, 1937). However, people’s preferences typically deviate from this prescription by showing steeper discounting over short delays than over long ones (e.g., Green & Myerson, 1996); this pattern is often labelled “hyperbolic”, but it has been modelled with a wide range of functions, each capturing distinct psychological insights (see Doyle, 2013, for a review).

The present experiments contribute to our understanding of intertemporal choice by examining people’s expectations about the options they will encounter in intertemporal choice tasks. For example, when asked: “Would you rather receive $10 now or $15 in... ”, what expectation do decision-makers have about the to-be-revealed delay? And how does that expectation shape their subsequent evaluation of the options?

These questions are motivated by a long line of research showing that people internalize and exploit ecological regularities to make inferences when they have incomplete information (e.g., Brunswick, 1943; Gigerenzer et al., 1999; Skylark, 2018), and that recent experience with attribute values and trade-offs shapes the attractiveness of a given option (e.g., Stewart et al., 2003; Tversky & Simonson, 1993; Rigoli & Dolan, 2019). Most pertinent to the current work is a series of studies by Pleskac and colleagues investigating people’s expectations about the trade-off between risk and reward.

In an initial series of studies, Pleskac and Hertwig (2014) found that, across a wide range of real-world domains, larger rewards are associated with smaller probabilities, such that gambles gravitate towards being “fair bets”.^1^ Pleskac and Hertwig suggest that people have internalized this regularity and use it when confronted with incomplete information about a risky option – i.e., that people employ a “risk-reward heuristic”.^2^ They contrast this hypothesis with traditional economic theory, in which probability, money, and time are all independent factors (e.g., Savage, 1954), and with the predictions of a “desirability bias” wherein people optimistically rate desirable outcomes as more probable then undesirable ones (e.g., Krizan & Windschitl 2007).

Pleskac and Hertwig (2014) tested their hypothesis by presenting a lottery that costs $2 to play and which offers the opportunity to win $2.50, $4, $10, $50, or $100 (with the value varied between participants). As predicted, participants’ estimates of the probability of winning were negatively associated with the value of the prizes. Having made their estimates, participants were asked whether they would play the lottery: willingness to play was positively associated with both the stated prize and the inferred probability. Skylark and Prabhu-Naik (2018) subsequently found that people likewise infer missing reward values from stated probabilities; again, the stated and inferred values both predicted subsequent choice. More recent work by Leuker et al. (2018; 2019a,b) has directly manipulated the risk-reward trade-off in a learning environment and found that this affects subsequent inferences about, and choices between, risky options for which probability information is not provided.

We ask whether similar principles operate in the domain of intertemporal choice. A positive correlation between time and reward is found in many contexts. One simple justification is given by Stewart et al. (2006) and also follows from Pleskac and Hertwig’s (2014) studies of the ecological risk-reward relationship: large gains are much rarer than small ones, so the time between large gains must on average be longer. However, it is not clear that people will have internalized this association – and if they have, it is not clear that it will take the form of a single, simple delay-reward function, because in everyday life people will experience a variety of such functions. For example, in games of chance like roulette the expected waiting time for a given outcome is a linear function of the payoff for that outcome, whereas bank accounts often offer returns that grown exponentially via compound interest – although the interest rate may be higher for longer term investments (to compensate for increased risk) or may vary unpredictably.^3^ And investments based on stock prices, or other instruments, can show even more complex relationships between time and returns. Thus, we predict that a decision-maker who brings their past experience to bear in a psychological study of inter-temporal choice will expect a positive association between delay and reward, but given the variety of forms that they are likely to have encountered we remain agnostic about the precise form of this expectation.

The existence of such a “delay-reward heuristic” would be important for several reasons. First, it would support the general claim that humans internalize and exploit ecological structure when making decisions from incomplete information. Second, the beliefs that people hold about the probable delay-reward trade-off may cast new light on efforts to identify the best mathematical description of intertemporal choice data (e.g., McKerchar et al., 2009), because such data would reflect variable past experiences rather than an immutable discounting function. Third, the existence of a delay-reward heuristic could provide new insights into individual differences in delay discounting (e.g., Reimers et al., 2009): variation in environmental context will produce heterogeneous preferences, and if particular demographic or dispositional variables are associated with particular contexts then this could partly or wholly explain the relationship between these individual-difference variables and people’s intertemporal choice behaviour.

In four experiments, we adopt the approach of Pleskac and Hertwig (2014) and Skylark and Prabhu-Naik (2018) to investigate how changes in the stated delay affect inferences about the value of a monetary reward and vice-versa. We focus on the kinds of choice between immediate and delayed monetary outcomes with which we began this paper, and frame the task as being “a psychology experiment” because we believe that this is the most common context in which people encounter this kind of simplified decision.

## 2 Methods

The four experiments were similar so their Methods are described together. The study materials are available from https://osf.io/e2fnt/

### 2.1 Participants

Studies 1A and 1B recruited US-based participants via Amazon’s Mechanical Turk (www.mturk.com); Studies 2A and 2B recruited UK-based participants via Prolific (www.prolific.co). In all studies, eligible participants were those who provided complete data, who were aged 18+, who did not self-report past participation, and whose IP address had not previously occurred in the data file of the current study or earlier in the study series (including in related studies not reported here). Full details of the screening/eligibility requirements are given in Appendix 1. The final samples are described in Table 1.

### 2.2 Design and Procedure

Studies 1A and 1B were exploratory; Studies 2A and 2B were pre-registered confirmatory studies (https://aspredicted.org/mn3vn.pdf). All studies were conducted online. After an initial landing page, information sheet, and consent form, participants were told that they would be asked to consider a simple financial decision. They were told that although the scenario was hypothetical, they should answer as honestly and accurately as they could and that there were no right or wrong answers.

#### 2.2.1 Study 1A

In Study 1A, participants were randomly assigned to 1 of 6 conditions, which differed in the time until the delayed reward. In the “1 day” condition, participants were told:

Suppose that you take part in a Psychology experiment in which the experimenter offers you a choice between two financial options. Due to a random printing error, part of one of the options is missing, so you can’t see the value that is meant to be displayed. The choice that the experimenter presents you with is shown below, with the missing value replaced by an “X”.

Which would you choose?

Option A: Receive $10 now

Option B: Receive $X in 1 day’s time

What do you think the missing value, X, is? Enter a number in the box below.

There followed a text box in which participants entered their judgment (only numeric responses were permitted). Note that they did not make a choice at this point. On the page after the estimation question, participants were told:

Suppose that your estimate of the missing value is correct. That is, suppose that the experimenter is offering you a choice between:

Option A: receive $10 now

Option B: receive [participant’s estimate] in 1 day’s time

Which would you choose?

Participants indicated their choice by selecting between 2 radio buttons labelled “Option A” and “Option B”. The 1 week, 2 weeks, 1 month, 6 months, and 1 year conditions were identical to the 1 day condition, except that the corresponding time period was used when stating the delayed option; each participant completed a single condition (i.e., made one estimate followed by one choice).

Finally, participants were asked whether they had previously started or completed the survey, and for their age and gender (male, female, or prefer not to say). After answering these questions, participants proceeded to a debriefing sheet. All questions required a response before the participant could progress to the next page.

##### 2.2.2 Study 1B

Study 1B was identical to Study 1A, but the estimation task specified the delayed amount as either $13, $18, $23, $28, $33, or $38, and the missing value “X” referred to the delay associated with that outcome (e.g., the choice was: “Option A: Receive $10 now. Option B: Receive $13 in X”). Participants indicated their estimate of X (which they were told could include a decimal point) via a text box and selected one of 4 radio buttons to indicate the temporal units (“Day(s)”, “Week(s)”, “Month(s)”, or “Year(s)”). As in Study 1A, they then proceeded to a screen that asked them to suppose that their estimate was correct and asked them to choose between the smaller-sooner and larger-later rewards, with their estimate of the delay used for the delayed option.

##### 2.2.3 Studies 2A and 2B

Studies 2A and 2B were identical to Studies 1A and 1B, respectively, except as follows. All monetary values were in Sterling (£) and delays were expressed in days: in Study 2A, participants were randomly assigned to one of 8 delays: 1, 4, 12, 27, 63, 88, 122, or 243 days, and estimated the reward; in Study 2B, participants were assigned to one of 8 future rewards: £11, 13, 15, 18, 23, 29, 38, or 54, and estimated the delay in days. Both studies included 2 multiple-choice attention-check questions after participants had made their choice; one question asked the value of the immediate reward (correct answer: £10); the other question asked why one value was originally replaced by an “X” (correct answer: because of a printing error). Participants who answered both questions correctly were labelled “Attentive”. Finally, we added a question that probed the participant’s past encounters with intertemporal choices studies [“Have you ever previously taken part in a Psychology study in which you were asked to choose between two amounts of money that differ in when they would be given to you (i.e., like this study)?”; response options: Yes/No/Don’t know]. Participants who answered “No” were labelled “Novices”. Studies 2A and 2B were run in parallel; participants were randomly assigned to one or the other.

#### 2.3 Data treatment

We pre-registered that we would exclude any negative estimates, but there weren’t any. All time values were converted to “days” (assuming 365 days per year and 365/12 days per month; in Study 1A, the delay for the “6 month” condition was treated as 365/2 days). For regression analyses, stated delay and stated reward were each divided by 10 (so the coefficients represent the effect of a 10-day or 10-dollar change), and age was divided by 10 and mean-centred (so the coefficients represent the effect of being 10 years older than average). Gender was coded -0.5, 0.5, and 0 for females, males, and “prefer not to say”, respectively.

## 3 Results

For Studies 1A and 1B, we began with simple tests of whether estimated rewards and delays depend on stated delays and rewards, and of whether estimates and stated values both influence choices. We then conducted more comprehensive exploratory analyses and robustness checks, which we subsequently pre-registered as the analysis plan for Studies 2A and 2B.

For Studies 1A and 1B, *p*-values less than .05 were treated as potentially important and we computed 95% confidence intervals; for the pre-registered Studies 2A and 2B, alpha was set to .01 and we report 99% confidence intervals.

A small proportion of participants inferred future rewards that were smaller than the immediately-available amount. Because participants were told there were no right or wrong answers, and because some may have believed they would be asked about a negative trade-off, we report the results with all estimates included in the analyses.

### 3.1 Studies 1A and 1B

**Figure 1:**
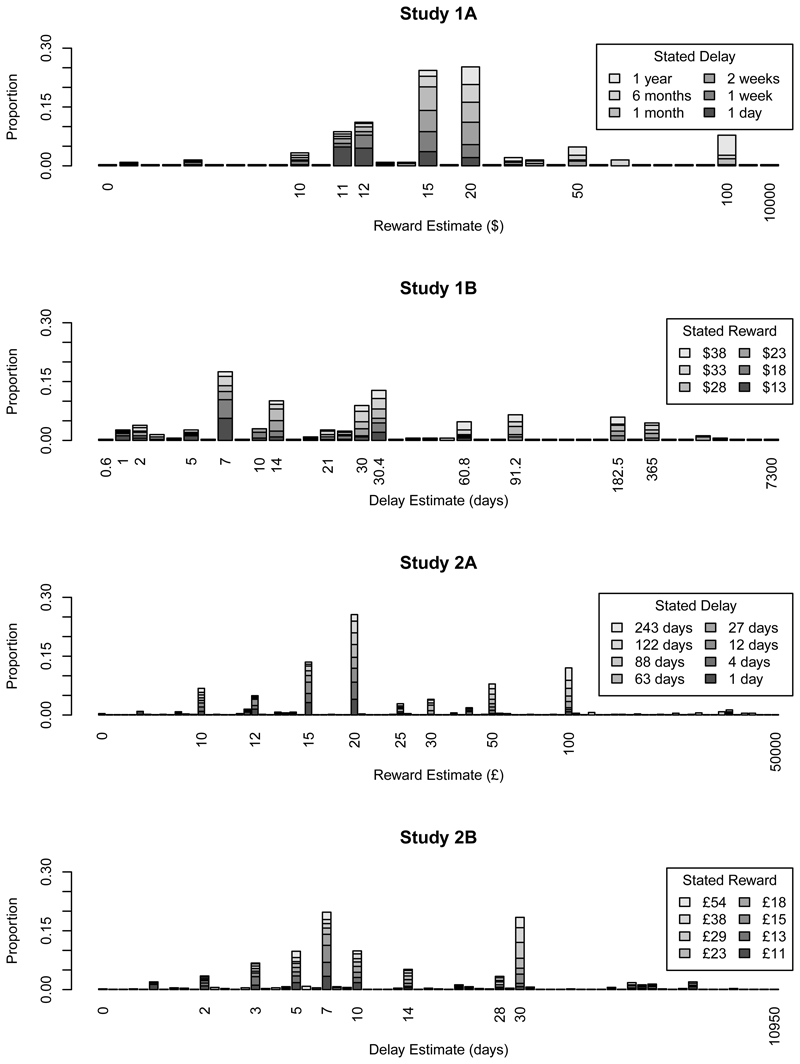
Unique responses in each experiment. In all studies, participants tend to use just a handful of response values when inferring rewards or delays, although this is against a background of more idiosyncratic estimates. The numbers below the x-axis label the most popular values, along with the smallest and largest estimate in each study. Note that the x-axis is ordinal: the values are simply arranged from smallest to largest.

In all studies, estimates (inferred rewards and delays) were highly positively skewed but approximately normal after transformation by *log*_10_(*x* + 1). The normality was only approximate: participants typically selected from a small set of possible values [for example, 6 months (182.5 days) when estimating the delay in Study 1B]. Figure 1 shows, in ascending order, the unique responses in each study; the y-axis shows the proportion of participants in each study who made that response, with data from each condition indicated by different shades of grey. This strong tendency to round numeric values is common in estimation tasks (e.g., Laming, 1997; Matthews & Stewart, 2009; Matthews et al., 2016); we discuss its implications below.

#### 3.1.1 Basic analyses

**Figure 2:**
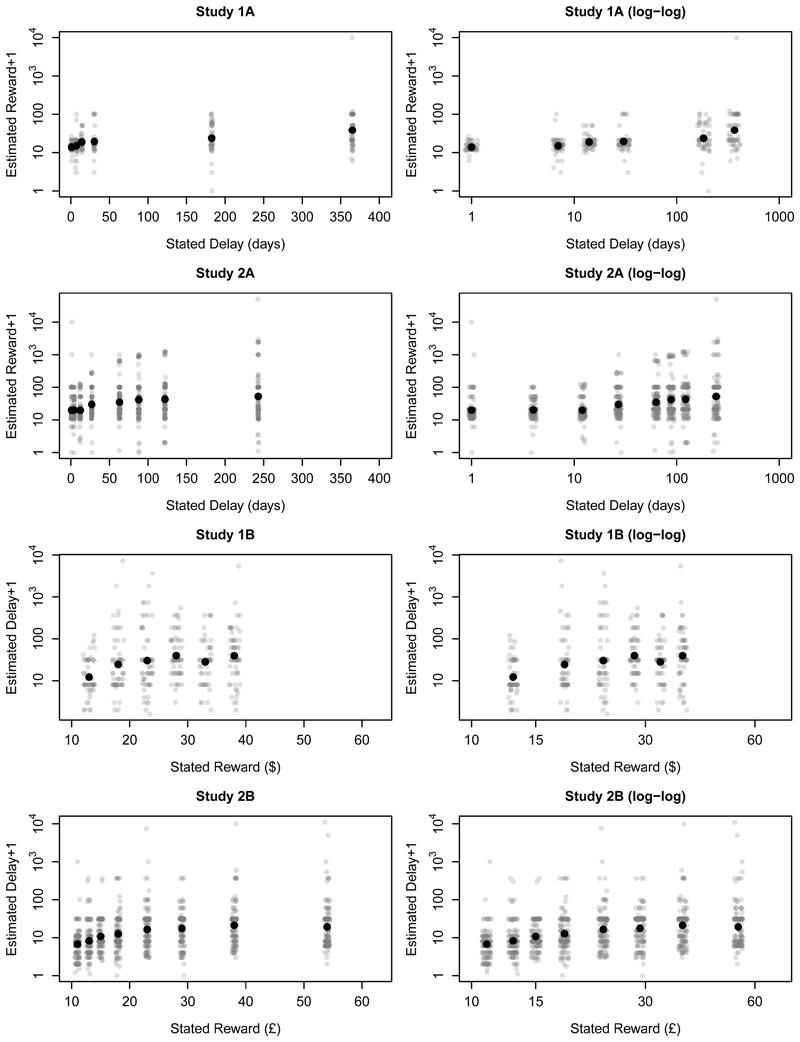
Estimated rewards as a function of stated delays, and estimated delays as a function of rewards. The plot shows *log*_10_(*estimate* + 1) against condition, with the y-axis tick marks exponentiated to improve clarity. Values have been jittered to reduce over-plotting. The right-hand panels show the same data with a logarithmic x-axis.

**Figure 3:**
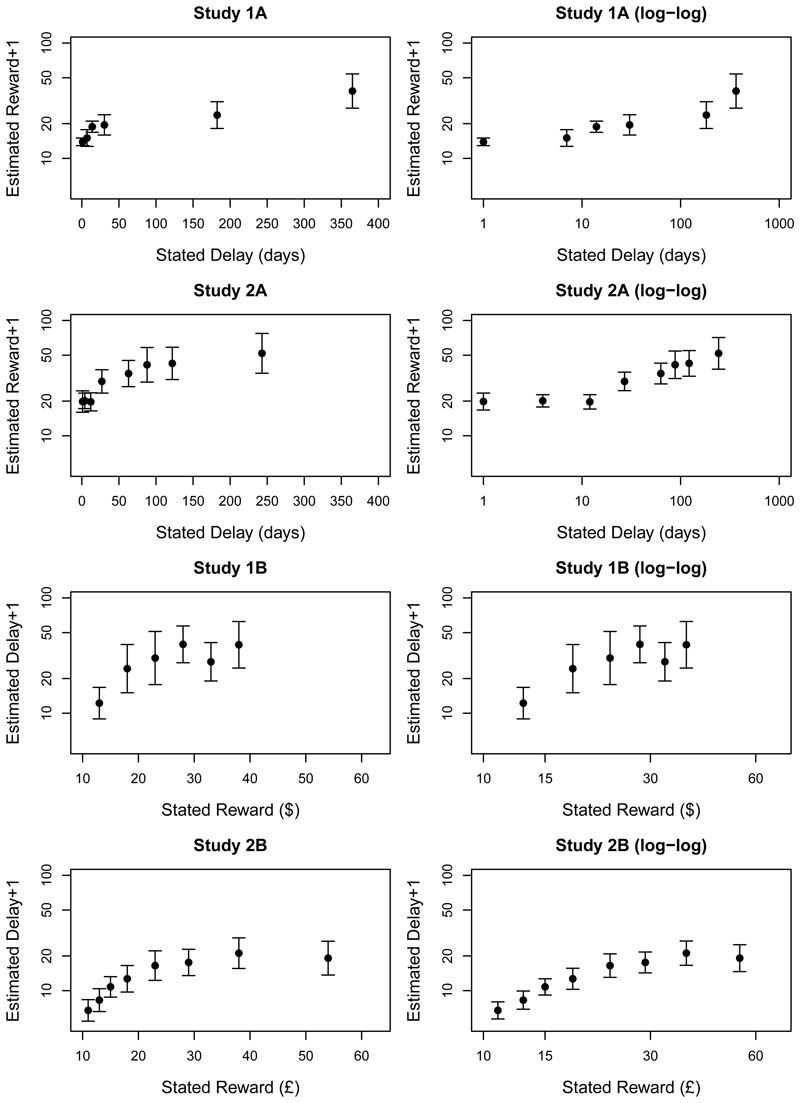
Figure 2 re-plotted without the raw data points. Error bars show confidence intervals (95% for Studies 1A and 1B, 99% for Studies 2A and 2B).

Table 2 shows descriptive statistics for each study. Figure 2 plots estimates against conditions, with solid points indicating the means. (For this plot, we log-transformed the estimates after adding 1 to deal with zeroes. To improve clarity, we transformed the y-axis tick marks as 10^*value*^; thus, the y-axes are labelled "Estimate + 1".) The right-hand panels show the same data with a logarithmic x-axis, which helps to clarify the effects for low values of the stated delay/reward. The presence of some extreme responses compresses the bulk of the data, so Figure 3 re-plots the means with confidence intervals, making the pattern clearer. The impression from the figures is that inferred delays increased with stated rewards, and vice-versa. Kruskall-Wallis tests confirmed that the stated value of the delay affected estimates of the reward in Study 1A, *χ* ^2^(5) = 75.08, *p* < .001, and that stated rewards affected estimates of the associated delay in Study 1B *χ* ^2^(5) = 28.67, *p* < .001. One-way ANOVAs on the log-transformed estimates led to the same conclusions [Study 1A: *F*(5, 327) = 15.56, *p* < .001, *η* ^2^ = .192; Study 1B: *F*(5, 331) = 5.21, *p* < .001, *η* ^2^ = .073].

**Figure 4:**
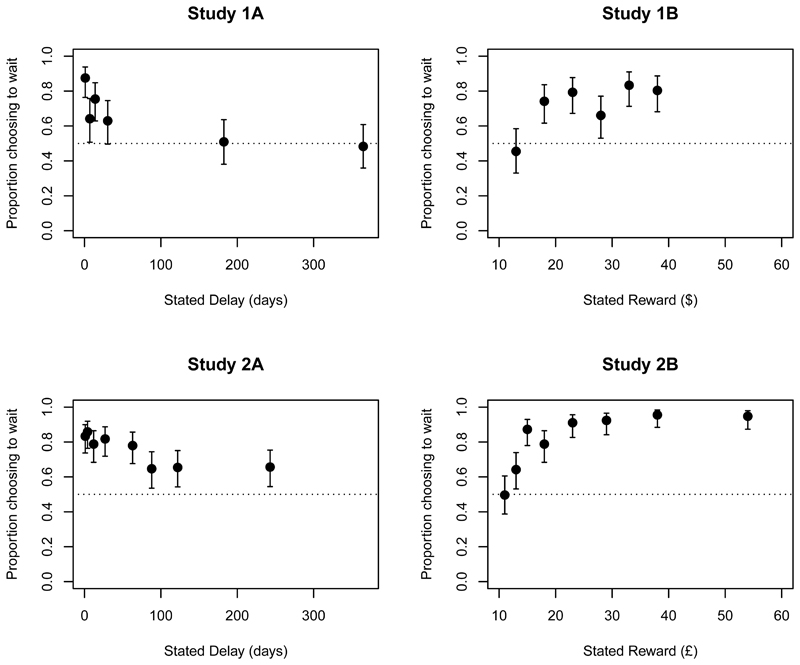
Choice of delayed option as a function of stated delay and stated reward. Error bars are Wilson confidence intervals (95% for Studies 1A and 1B; 99% for Studies 2A and 2B). The dotted line indicates indifference.

Figure 4 shows the proportion of participants choosing to take the delayed option in each condition. If participants’ inferred delays or rewards corresponded to their indifference points (i.e., if they thought they were going to be offered options for which the immediate and delayed options were equally attractive), the data points would fall on a flat line at 0.5. Clearly, this is not the case: in all studies, there is an overall tendency to choose the delayed option. And at the group level, the data in Figure 4 suggest that larger stated delays led to less willingness to wait, implying that the increase in inferred reward was not sufficient to offset the increase in waiting time. Similarly, increases in stated reward led to increased willingness to wait, implying that the increase in inferred delay was not sufficient to offset the increasing appeal of the potential gain.

These effects are further evidenced by Table 3, which shows the results of logistic regression of choice (immediate reward = 0; delayed reward = 1) on condition (stated delays and rewards, treated as continuous variables) and log-transformed estimates (inferred rewards and delays). In both studies, there was a positive association between willingness to wait and the stated or inferred reward, and a negative association between willingness to wait and stated or inferred delay. The effects of estimate remain after controlling for condition: for a given stated delay, people who inferred a larger future reward were more likely to choose to wait; and for a given stated reward, those who inferred a larger delay were less inclined to wait.

#### 3.1.2 The effects of age and gender on inferences

We next conducted exploratory analyses to investigate whether age and gender predict inferences and choices. First, we computed the Kendall’s correlation matrices shown in Table 4. In Study 1A, estimated rewards were smaller for men than for women and in Study 1B estimated delays were larger for older participants. These associations are complicated by the fact that, in both studies, age and gender are confounded.

To clarify the picture, we regressed log-transformed estimates on age, gender, and condition. We ran several versions of the analysis to help ensure that our results were not a consequence of particular analytic decisions. In one version, we treated condition as a continuous predictor, as above. However, because the relationship may not be linear, we ran a separate version with condition as a categorical predictor with successive-difference contrast coding, such that the coefficients test the difference between each adjacent pair of stated delays or rewards; in the case of categorical coding, we tested the overall effect of condition by using an F-test to compare the model that included condition with one that did not. We ran both the continuous-condition and the categorical condition analyses twice: once with ordinary least squares (OLS) regression and once with robust regression using the lmrob function from the robustbase package for R (Maechler et al., 2020). We report the OLS analysis with condition as a factor in the main text, and note any discrepancies between these results and the alternative analyses – whose results are reported in full in the Supplementary Materials (https://osf.io/e2fnt/).

Table 5 shows the results for Studies 1A and 1B. Consistent with the earlier analyses, estimated rewards are positively associated with stated delays (Study 1A), and estimated delays are positively associated with stated rewards (Study 1B). In addition, estimated rewards are negatively associated with age in Study 1A and estimated delays are positively associated with age in Study 1B. The analysis also suggests that, in Study 1B, males inferred longer delays than did females.

The pattern of results was identical for all other versions of the analyses, except that the tendency for male participants to infer lower rewards than female participants in Study 1A had a 95% CI that excluded zero in the robust regression analysis with condition treated as a continuous variable (b = −0.054 [−0.105, −0.003], p = .039).

#### 3.1.3 The effects of age and gender on choice

Finally, we took a similar approach to exploring the effects of demographic variables on decision-making by regressing choice on condition, estimate, age, and gender. Again, we conducted several versions of the analysis, to check robustness: for each study we ran 8 versions formed by factorially varying (a) whether condition was coded as a continuous or categorical predictor, (b) whether estimates were entered as raw responses or log-transformed values, and (c) whether conventional or robust logistic regression was used, with the latter implemented via the glmrob function in the robustbase package for R using the recommended “KS2014” setting.

Table 5 shows the results of the standard regression with condition as a categorical variable and using log-transformed estimates. As expected from the previous analysis, willingness to wait was positively associated with stated and inferred reward, and negatively associated with stated and inferred delay. Neither age nor gender was appreciably associated with choice behaviour. All of the other versions of the regression analyses yielded the same conclusions

### 3.2 Studies 2A and 2B

For Studies 2A and 2B, we pre-registered an analysis plan that comprised all of the analyses from Studies 1A and 1B that incorporated age and gender. We also pre-registered that we would apply all analyses to 3 different versions of the datasets: the full sample; only those participants who passed both attention-checks; and only those attentive participants who also indicated that they had never previously taken part in a monetary intertemporal choice experiment (“attentive novices”). We report the results for the full sample using the same regression output as for Studies 1A and 1B, and again note any differences between these and other versions of the analyses (which are reported in full in the Supplementary Materials). In the pre-registration, we predicted that the effects of condition on estimates and of condition and estimates on choice would match those found in Studies 1A and 1B, and that older participants would infer longer delays and smaller rewards than younger participants.

As before, descriptive statistics are shown in Table 2; Figure 1 illustrates the tendency of participants to use a small set of round response values; Figures 2 and 3 show the estimates for each condition; and Figure 4 plots the proportion of participants who chose to wait in each condition. The Kendall correlations are again shown in Table 4; the regression results are shown in Table 6.

The results of both studies are very similar to Studies 1A and 1B, and match the predictions. In Study 2A, participants inferred larger rewards for longer stated delays, and younger participants inferred larger rewards than did older ones, with no meaningful effect of gender. Willingness to choose the delayed option was greater for shorter stated delays and for larger inferred rewards, with no appreciable effect of age or gender. The results were the same for all samples and all regression specifications. In Study 2B, participants inferred larger delays from larger stated rewards, and older participants inferred longer delays than did younger ones. These results were the same for all samples and specifications. In the choice data, the willingness to wait was positively associated with stated reward and negatively associated with inferred delay, with no effect of age or gender. The only discrepancies across the 24 versions of the analysis were that in 6 cases the 99% CIs for the effect of inferred delay included zero; in all of these cases the coefficients for the estimated delay were again negative, and the CIs only just graze zero.^4^

Taken together, the results of Studies 2A and 2B replicate the patterns found in Studies 1A and 1B.

## 4 Discussion

We found that: (a) participants typically inferred larger monetary rewards from longer stated delays, and vice-versa; (b) estimates of delay and reward were positively skewed and usually took a relatively small number of distinct values; (c) willingness to wait was positively correlated with stated and estimated rewards, and negatively associated with stated and estimated delays; and (d) that older participants inferred longer delays and smaller future rewards than did younger participants, but did not differ in their willingness to wait for the inferred delayed option. We discuss these results and outline future research directions.

### 4.1 Where do the inferences come from?

Our results are consistent with the idea that people approach decisions with prior expectations about the likely trade-off between attributes, and that these expectations shape subsequent decisions (Pleskac & Hertwig, 2014; Tversky & Simonson, 1993). It is important to consider the possible origins of participants’ numerical estimates in more detail. One basic distinction is between an associative strategy and a matching strategy. Under the former, our participants encoded past pairings of attribute values and used the provided value of one attribute (e.g., time) to retrieve associated values of the other (e.g., monetary reward). In contrast, magnitude-matching involves producing an estimate for the missing attribute that is subjectively-equal to the stated attribute (e.g., responding with a monetary value that “feels the same” as the stated delay – for example, by choosing a value that has the same rank position in a contextual set of memory items; e.g., Stewart et al., 2006). Under the associative account, previously-encountered pairings of attributes are critical; in the magnitude-estimation account, only the values within a given attribute dimension are important. The two possibilities could therefore be distinguished by constructing training environments which present the same temporal and monetary values in different pairs (cf Leuker et al., 2018).

A second distinction contrasts the use of purely within-option information with the use of between-option information. In the case of an associative strategy: people might base their inferences on simple time-money pairings, using the stated reward to “pull out” a likely delay; or they might draw upon the particular combinations of monetary and temporal values that defined the pairs of options in previous choice tasks – a strategy which would permit different inferences about the probable size of a future reward depending on the value and timing of the more immediate option. Likewise, a participant who employs a magnitude-matching approach might simply seek a monetary value that has the same subjective magnitude as the stated delay; or they might seek a monetary value such that the difference between this reward and that of the immediate option matches the difference between the time of the delayed option and “now”. In studies like ours, the within-option strategy would mean that people make the same inferences about the missing values irrespective of the value of the immediate reward, a possibility which could readily be tested in future. It is quite possible that different inference/estimation strategies might be used in different contexts.

An alternative approach would be to consider a decision-maker’s reasoning process when confronted with these kinds of choices. Decision-makers may expect larger delays to be associated with larger rewards because that would be necessary to make options with a range of delays equally attractive on average. For example, in a financial product marketplace, rewards would have to be greater for long-delay products in order for them to compete with short-delay products.^5^

### 4.2 Implications for theories and studies of time preference

Mathematical models of delay discounting are usually interpreted in terms of the psychological representation of time and money and their interaction. For example, the popular generalized hyperbolic function (Myerson & Green, 1995) is taken to indicate power-law scaling of money coupled with a focus on rate of reinforcement (Doyle, 2013; Green & Myerson, 1996). Our results suggest that choices reflect the interplay between the monetary and time values comprising each option and the decision-makers’ expectations about those values. In particular, people may make choices on the basis of whether an offered option seems to be good value relative to their expectation of the trade-off between delay and reward.

This could lead to substantial reinterpretation of existing functions; it could also lead to alternative models. An obvious step towards the latter would be a mathematical characterization of the “inference functions” that map stated values of time and money into expectations about missing values of money and time. Indeed, we originally hoped that the current studies would provide a first step in this direction; for example, we experimented with fitting common discounting functions to the estimates from Studies 1A and 1B plotted in Figures 2 and 3. However, although it is possible to fit candidate functions to our participants’ data, we ultimately decided it would be unwise because the strong tendency of participants to use “round numbers” means that conventional continuous functions are inappropriate. Although the preference for round numbers is widespread (e.g., Laming, 1997; Matthews & Stewart, 2009), the resulting almost-discrete distributions are not well-characterized, so modelling is a challenge. One open question is whether the limited set of monetary and time values produced by our participants reflects a response tendency or a genuine expectation that the options will be familiar, round numbers. The latter possibility might have implications for studies that employ non-round numbers (e.g., Kirby et al., 1999).

**Figure 5:**
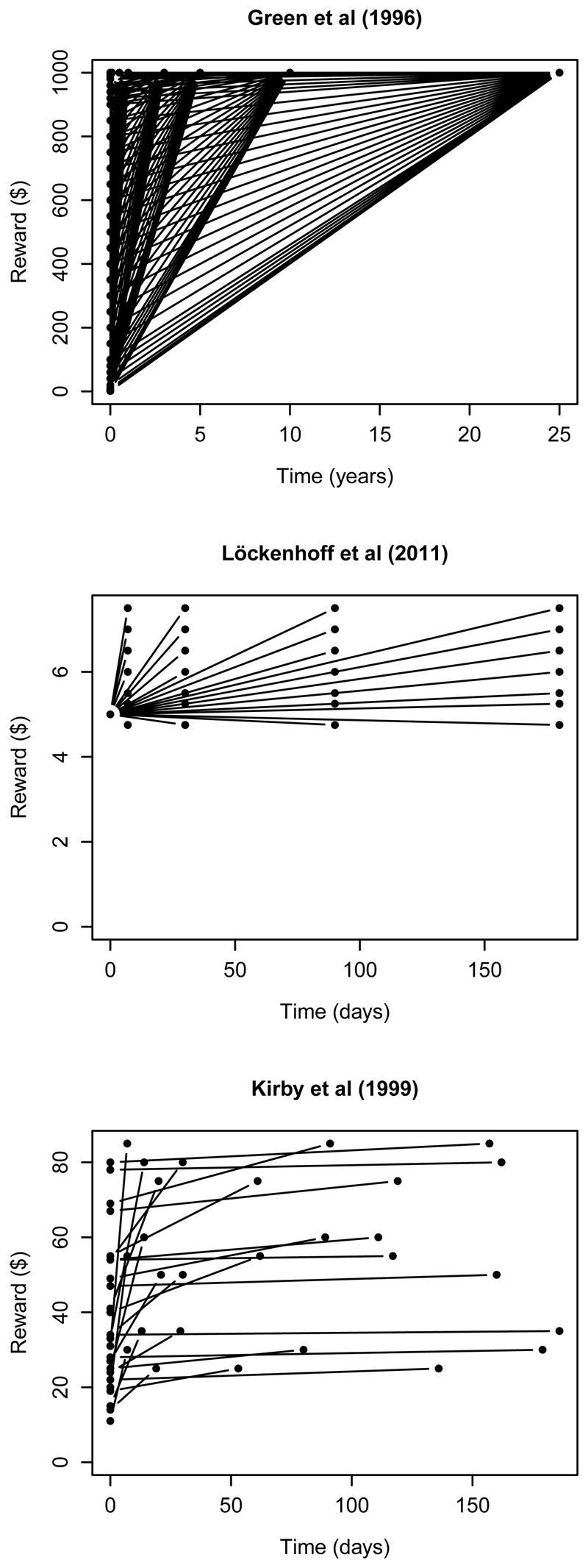
Trade-offs between money and time in typical experiments of intertemporal choice.

Our results also speak to the methodologies used to study intertemporal choice. Some studies use a single pair of options, or employ an adaptive procedure such that presented trade-offs depend on prior choices and are thus idiosyncratic to the participant, but many studies present a substantial fixed set of options. One common approach is to fix one monetary value (e.g., the delayed reward) and offer a set of possible values for the other, and then to repeat this for a range of delays. Two examples are shown in the top 2 panels of Figure 5. Because the same set of rewards is used for all delays, such studies offer steeper delay-reward trade-offs for longer delays than for shorter ones. (Many other papers use the same kind of approach with different specific values; see e.g., Mahalingham et al., 2014; Shamosh et al., 2008). The widely-used “monetary-choice questionnaire” (Kirby et al., 1999; Towe et al., 2015) uses a more diverse mixture of monetary and temporal values, but the options again involve steeper trade-offs when delays are short (bottom panel of Figure 5). Presumably these patterns reflect researchers’ intuitions that participants will show hyperbolic-like discounting. From our perspective, people’s choices in such tasks will (at least partly) reflect the discrepancies between the lines plotted in Figure 5 and the curve describing participants’ expectations about the delay-reward trade-off (Figures 2 and 3). Moreover, we would expect participants to update their expectations in light of the options they have encountered earlier in the session – so the curves shown in Figure 5 might shape, not simply measure, participants’ discounting functions (see Stewart et al., 2015, for similar ideas, and Alempaki et al., 2019, Matthews, 2012, for limits on their scope).

### 4.3 Implications for individual differences in intertemporal choice

Our studies suggest that part of the reason people differ in their preferences is because they have different expectations about the delays and rewards that they will encounter – presumably because of different past experiences with the delay-reward structure of their environment. Beyond offering a possible explanation for unexplained variance in choice behaviour (Myerson et al., 2016), this suggests a new perspective on previously-reported associations between inter-temporal choice preferences and a variety of demographic and dispositional variables (e.g., Du et al., 2002; Mahalingham et al., 2014; Reimers et al., 2009). In particular, we found that older adults expected smaller future rewards and longer delays than did younger adults. The absolute value of the effect was not large, but to the extent that older people typically have more pessimistic expectations about delayed rewards than do young people, they will be more pleasantly surprised (or less unpleasantly surprised) by any offered “larger-later” option – and hence presumably more likely to choose it. Several studies have indeed found that older people are more likely to choose to wait in the kind of monetary-choice experiment used here (e.g., Green et al., 1994; Jimura et al., 2011; Li et al., 2013; Löckenhoff et al., 2011; Reimers et al., 2009; Whelan & McHugh, 2009), leading to claims that “delay discounting declines across the lifes-pan” (Odum, 2011, p. 6). However, other studies have found the opposite effect (e.g., Albert & Duffy, 2012) a curvilinear effect of age (e.g., Richter & Mata, 2018), or no association (e.g., Chao et al., 2009; see Löckenhoff & Samanez-Larkin, 2020). Therefore, rather than making a strong claim about the association between age and time-preference, we simply raise the possibility that variables found to predict temporal discounting may do so partly via effects on expectations about the delay-reward trade-off. (It should also be noted that caution is necessary when interpreting age effects found in volunteer samples such as ours. It is possible, for instance, that people volunteer for different reasons at different ages and some other unobserved variable is driving the observed differences.)

### 4.4 Future Directions

Our results suggest several lines of future work, including. . .

1. Retaining ambiguity about the unknown values. In our studies, participants were asked to assume that their estimate of the missing delay or reward was correct, such that the choice task used their estimate to form the delayed option. Resolving the ambiguity in this way helps to clarify the relationship between people’s inferences about the missing attributes and their indifference points. However, outside the lab people often have to make choices when the delay or reward remain ambiguous (Dai et al., 2019), and it would be useful to explore the links between stated values, inferred values, and choice behaviour in that kind of situation. This would also permit investigation of order effects: our design necessitated that estimates came before choices, but with ambiguous options task order could be counterbalanced to see whether (for example) the act of choosing affects inference, and vice-versa.
2. Investigating inferences and choices in a within-subject design. We focused on “one-shot” decisions so as to avoid participants constructing inferences based on the local environment of the test session (Pleskac& Hertwig, 2014; Skylark & Prabhu-Naik, 2018) but, as noted above, studies of time preference often try to elicit individual discounting functions by presenting people with a set of choices, and generalizing our approach to this paradigm may be profitable.
3. Testing whether environmental contingencies underlie inferences about missing attributes by manipulating the delay-reward trade-off in a training environment to see whether this directly modulates inferences about missing attributes, and choices when all attributes are present, in a subsequent test stage – as has been done for studies of the risk-reward heuristic (Leuker et al., 2018, 2019a,b).
4. Generalizing to other contexts. We focused on expectations about the delay-reward trade-off in psychology research. Although researchers are typically interested in “real” decisions, simplified money-time trade-off questions are used as a testbed for developing and testing theories of intertemporal choice, so understanding the role of expectations in this context is important. Nonetheless, it will be necessary to generalize to other scenarios – for example, by asking people to infer the final value of a fixed-term investment fund, or to estimate the price of an “express delivery” service that brings forward the point at which a product can be consumed.
5. Examining whether other individual difference variables predict inferences about missing attributes. Of particular interest are variables such as socio-economic status and the “Big 5” personality traits, which have been associated with patterns of intertemporal choice (Mahalingham et al., 2014; Oshri et al., 2019). Does this reflect different expectations about the delay-reward trade-off? And do differing expectations themselves reflect different environmental experiences, as might be expected for demographic variables such as income?
6. Examining the consequence of violated expectations. Our choice task focused on participants’ willingness to wait for the delayed option that they had inferred from the stated values. A straightforward extension would be to present options that deviated from the participant’s inference. The simple prediction is that participants who inferred/expected longer delays will be more likely to choose to wait than those who inferred short delays – and vice-versa for inferred rewards.
7. Omitting more information. Choices often involve ambiguity about more than one attribute value. For example, both the shorter and longer delay may be unknown. We can envisage studies that probe expectations about missing values from progressively diminished information, including, in the limit, inferences about the delay-reward trade-off when all that is known is that there are two options that differ in when they occur. Such studies would establish more comprehensively the background knowledge that participants bring to time-preference tasks, and how they construct expectations using this knowledge in combination with the information provided in the task.

We have collected preliminary data to explore some of these issues (details are available from the corresponding author), but there is much more to do in future.

## 5 Conclusions

These studies indicate the potential value of probing people’s expectations about the trade-off between time and money. Despite widespread individual variation, there were reliable tendencies to infer longer delays from larger rewards, and vice-versa, with implications for theories and empirical investigations of delay discounting. In addition, age was somewhat associated with more pessimistic expectations, such that these expectations may partly explain previously-observed differences in discounting by older and younger adults.

## Supplementary Material

Appendix

## Figures and Tables

**Table 1:** Demographic data.

	Study 1A	Study 1B	Study 2A	Study 2B
N	333	337	1074	1064
Age range; M (SD)	20−74; 35.9 (11.5)	19−72 (10.2)	18−87; 35.9 (13.7)	18−82; 35.5 (13.0)
Gender: M, F, prefer not to say	193, 140, 0	200, 137, 0	334, 734, 6	331, 730, 3
Attentive	—	—	844 (78.6%)	833 (78.3%)
Novice	—	—	814 (75.8%)	781 (73.4%)
Attentive & Novice	—	—	638 (59.4%)	613 (57.6%)

Note: The “Attentive” row indicates the size and proportion of the sample that passed both attention-check questions; “Novice” indicates the size and proportion of sample who indicated that they had not previously taken part in a psychology study involving intertemporal choice between monetary outcomes.

**Table 2:** Descriptive statistics for inferred rewards and inferred delays.

**Study 1A**								
Delay	N	M	SD	95% CI	Med	LQ,UQ	GeoM	95 % CI
1 day	56	13.3	3.6	12.4, 14.3	12	11, 15	12.9	12.0, 13.8
1 week	53	16.6	14.7	12.6, 20.7	15	12, 18	13.8	11.8, 16.2
2 weeks	57	19.3	9.6	16.8, 21.8	15	15, 20	17.8	16.0, 19.6
1 month	54	24.3	25.2	17.4, 31.2	15	15, 20	18.3	15.1, 22.0
6 months	55	31.4	25.8	24.4, 38.3	20	15, 50	22.7	17.8, 28.9
1 year	58	219.5	1307.3	−124.3, 563.2	25	20, 100	36.8	27.2, 49.9
**Study 1B**								
Reward	N	M	SD	95% CI	Med	LQ,UQ	GeoM	95 % CI
$13	55	19.5	24.1	13.0, 26.0	7	7, 30.2	10.5	7.7, 14.4
$18	58	207.6	980.3	−50.2,465.3	14	7, 30.4	22.0	14.1, 34.3
$23	58	184.9	545.6	41.4, 328.3	20.5	7.3, 83.6	27.1	16.5, 44.2
$28	56	80.1	112.5	50, 110.3	30.4	14, 91.3	37.7	27.0, 52.5
$33	54	58.8	93.6	33.2, 84.3	30	14, 56.1	25.8	18.0, 37.0
$38	56	175.9	730.8	−19.8, 371.6	30.4	14, 91.3	36.4	23.7, 55.9
**Study 2A**								
Delay	N	M	SD	99% CI	Med	LQ,UQ	GeoM	99 % CI
1 day	138	101.6	853.1	−88.1, 291.3	15	12, 20	18.8	15.3, 23.1
4 days	134	23.7	19.8	19.2, 28.2	20	15, 20	19.1	16.4, 22.1
12 days	132	25.7	27.2	19.5, 31.9	15	12, 20	18.3	15.3, 21.9
27 days	137	52.3	101.1	29.7, 74.8	20	15, 50	28.6	22.9, 35.7
63 days	136	78.0	163.4	41.4, 114.6	22.5	20, 50	32.8	25.3, 42.4
88 days	133	129.8	261.5	70.6, 189.1	25	20, 100	40.3	29.1, 55.6
122 days	133	122.9	280.4	59.4, 186.5	30	20, 100	40.0	29.2, 54.8
243 days	131	585.7	4384.6	−415.8, 1587.1	50	20, 100	48.6	32.9, 71.6
**Study 2B**								
Reward	N	M	SD	99% CI	Med	LQ,UQ	GeoM	99 % CI
£11	133	15.4	86.7	−4.3, 35.0	7	3, 10	5.3	4.1, 6.7
£13	134	15.7	42.7	6.1, 25.4	7	3, 10	6.8	5.3, 8.7
£15	133	17.0	40.5	7.8, 26.2	7	7, 20	9.4	7.7, 11.6
£18	132	28.3	69.0	12.6, 44.0	10	7, 28	11.7	8.9, 15.2
£23	134	89.9	652.2	−57.3, 237.2	14	7, 30	14.8	11.1, 19.7
£29	131	30.7	55.0	18.1, 43.2	28	7, 30	16.6	12.8, 21.3
£38	134	118.0	864.7	−77.2, 313.2	28	7, 30	19.4	14.4, 26.0
£54	133	164.1	1042.1	−72.1, 400.2	10	6, 30	17.4	12.6, 24.0

Note: Med = Median; LQ = lower quartile; UQ = upper quartile; GeoM = geometric mean, calculated as [10^*log*_10_(*x*+1)^] – 1.

**Table 3:** Logistic regression of choice on stated value and inferred value.

**Study 1A**					
	b	SE	95% CI	*z*	*p*
Intercept	0.981	0.152	0.684, 1.279	6.463	<.001
Stated Delay	−0.034	0.008	−0.050, −0.017	4.012	<.001
Overall model: *χ* ^2^(1) = 16.37, *p* < .001, Pseudo-*R* ^2^ = .066					
Intercept	−1.338	0.569	−2.453, −0.224	2.354	.019
Est Reward	1.520	0.441	0.655, 2.385	3.444	.001
Overall model: *χ* ^2^(1) = 14.24, *p* < .001, *R* ^2^ = .058					
Intercept	−2.644	0.683	−3.983, −1.306	3.873	<.001
Stated Delay	−0.067	0.011	−0.089, −0.045	5.373	<.001
Est Reward	3.075	0.572	1.953, 4.196	5.890	<.001
Overall model: *χ* ^2^(2) = 56.13, *p* < .001, Pseudo-*R* ^2^ = .213					
**Study 1B**					
	b	SE	95% CI	*z*	*p*
Intercept	−0.407	0.376	−1.144, 0.330	1.082	.279
Stated Reward	0.540	0.149	0.247, 0.832	3.612	<.001
Overall model: *χ* ^2^(1) = 13.72, *p* < .001, Pseudo-*R* ^2^ = .057					
Intercept	1.509	0.308	0.906, 2.111	4.906	<.001
Est Delay	−0.402	0.189	−0.772, −0.031	2.126	.034
Overall model: *χ* ^2^(1) = 4.52, *p* = .033, Pseudo-*R* ^2^ = .019					
Intercept	0.190	0.427	−0.647, 1.028	0.445	.656
Stated Reward	0.664	0.159	0.352, 0.977	4.168	<.001
Est Delay	−0.617	0.202	−1.013, −0.220	3.046	.002
Overall model: *χ* ^2^(2) = 23.28, *p* < .001, Pseudo-*R* ^2^ = .096					

Note: The table shows the results of 3 different models for each study: one with stated value as the sole predictor; one with inferred (estimated) value as the sole predictor; and one with both predictors entered simultaneously. SE = standard error of coefficient. Est = Estimated. Pseudo-*R* ^2^ values are Nagelkerke’s *R* ^2^. Inferred values (estimates) were log-transformed as *log*_10_(*x* + 1).

**Table 4:** Kendall correlations

**Study 1A**			
	Est Reward	Stated Delay	Gender
Stated Delay	.378 (.001)		
Gender	−.116 (.016)	−0.079 (.104)	
Age	−.065 (.100)	.003 (.939)	−.138 (.003)
**Study 1B**			
	Est Delay	Stated Reward	Gender
Stated Reward	.195 (<.001)		
Gender	.074 (.109)	.025 (.607)	
Age	.205 (<.001)	−.039 (.334)	−.123 (.007)
**Study 2A**			
	Est Reward	Stated Delay	Gender
Stated Delay	.256 (<.001)		
Gender	.014 (.602)	.028 (.293)	
Age	−.117 (<.001)	.003 (.905)	−.032 (.208)
**Study 2B**			
	Est Delay	Stated Reward	Gender
Stated Reward	.254 (<.001)		
Gender	−.005 (.853)	.026 (.334)	
Age	.165 (<.001)	−.010 (.633)	−.027 (.293)

Note: Est = Estimated. Values in parentheses are p-values for the associated correlation.

**Table 5:** Regression results for Studies 1A and 1B.

**Study 1A**								
Estimates					Choices			
	b	95% CI	*t*	*p*	b	95% CI	*z*	*p*
Intercept	1.312	1.279, 1.345	78.422	<.001	−3.601	−5.127, −2.074	4.623	<.001
1w vs 1d	0.027	−0.087, 0.141	0.464	.643	−1.462	−2.459, −0.464	2.872	.004
2w vs 1w	0.089	−0.024, 0.202	1.547	.123	0.255	−0.603,1.113	0.582	.561
1m vs 2w	0.013	−0.099, 0.125	0.228	.820	−0.609	−1.457, 0.239	1.407	.159
6m vs 1m	0.097	−0.016, 0.21	1.676	.095	−0.936	−1.786, −0.086	2.158	.031
1y vs 6m	0.205	0.094,0.316	3.611	<.001	−0.687	−1.554,0.180	1.553	.121
Estimate					3.319	2.146, 4.493	5.543	<.001
Age	−0.035	−0.064, −0.006	2.394	.017	0.115	−0.112, 0.342	0.996	.319
Gender	−0.059	−0.126,0.008	1.726	.085	0.109	−0.415, 0.632	0.407	.684
Effect of condition: *F*(5, 325) = 15.43, *p* < .001					*χ* ^2^(5) = 57.66, *p*< .001			
Overall model: *F*(7, 325) = 12.4, *p* < .001, $R_{adj}^{2}=.194$					*χ* ^2^(8) = 72.57, *p* < .001, Pseudo-*R* ^2^ = .270			
**Study 1B**								
Estimates					Choices			
	b	95% CI	*t*	*p*	b	95% CI	*z*	*p*
Intercept	1.413	1.349, 1.476	43.779	<.001	2.208	1.48, 2.937	5.943	<.001
$18 vs $13	0.251	0.036, 0.467	2.292	.023	1.505	0.673, 2.338	3.544	<.001
$23 vs $18	0.066	−0.146, 0.278	0.613	.540	0.363	−0.534, 1.259	0.793	.428
$28 vs $23	0.149	−0.064, 0.363	1.370	.172	−0.611	−1.479, 0.257	1.380	.168
$33 vs $28	−0.123	−0.341, 0.094	1.110	.268	0.884	−0.038, 1.805	1.879	.060
$38 vs $33	0.176	−0.041, 0.394	1.588	.113	−0.034	−1.029, 0.961	0.067	.946
Estimate					−0.844	−1.291, −0.397	3.704	<.001
Age	0.185	0.123, 0.248	5.803	<.001	0.158	−0.111,0.426	1.151	.250
Gender	0.208	0.079, 0.337	3.170	.002	0.401	−0.135, 0.937	1.467	.142
Effect of condition: *F*(5, 329) = 5.54, *p* < .001					*χ* ^2^(5) = 33.68, *p* < .001			
Overall model: *F*(7, 329) = 9.65, *p* < .001, $R_{adj}^{2}=.153$					*χ* ^2^(8) = 40.52, *p* < .001 Pseudo-*R* ^2^ = .162			

Note: Inferred values (estimates) were log-transformed as log_10_(*x* + 1). $R_{adj}^{2}$ is adjusted *R* ^2^. Pseudo-*R* ^2^ values are Nagelkerke’s *R* ^2^. For row names in upper table: d = day, w = week(s), m = month(s), y = year.

**Table 6:** Regression results for Studies 2A and 2B.

**Study 2A**								
Estimates					Choices			
	b	95% CI	*t*	*p*	b	95% CI	*z*	*p*
Intercept	1.490	1.449, 1.530	94.614	<.001	−4.526	−5.794, −3.258	9.192	<.001
4d vs 1d	0.001	−0.148, 0.151	0.020	.984	0.105	−0.817, 1.026	0.293	.770
12d vs 4d	−0.002	−0.154, 0.149	0.041	.967	−0.440	−1.347, 0.467	1.250	.211
27d vs 12d	0.182	0.031, 0.332	3.114	.002	−0.300	−1.171, 0.571	0.888	.374
63d vs 27d	0.047	−0.103, 0.197	0.806	.420	−0.466	−1.332, 0.400	1.385	.166
88d vs 63d	0.084	−0.066, 0.235	1.446	.149	−0.954	−1.785,−0.122	2.955	.003
122d vs 88d	0.017	−0.134, 0.168	0.289	.772	−0.129	−0.953,0.695	0.403	.687
243d vs 122d	0.092	−0.060, 0.243	1.555	.120	−0.094	−0.948, 0.760	0.285	.776
Estimate					4.204	3.242, 5.166	11.260	.001
Age	−0.047	−0.074, −0.019	4.376	<.001	−0.023	−0.179, 0.133	0.378	.705
Gender	0.035	−0.046,0.117	1.116	.264	−0.006	−0.474, 0.461	0.034	.973
Effect of condition: *F*(7,1064) = 16.35,*p* < .001					*χ* ^2^(7) = 116.1, *p* < .001			
Overall model: *F*(9,1064) = 15.13, *p* < .001, $R_{adj}^{2}=.106$					*χ* ^2^(10) = 310.2, *p* < .001, Pseudo-*R* ^2^ = .374			
**Study 2B**								
Estimates					Choices			
	b	95% CI	*t*	*p*	b	95% CI	*z*	*p*
Intercept	1.127	1.088, 1.166	74.754	<.001	3.094	2.388, 3.800	11.288	<.001
£13 vs £11	0.073	−0.071, 0.217	1.301	.193	0.741	0.078, 1.404	2.880	.004
£15 vs £13	0.117	−0.027, 0.26	2.091	.037	1.493	0.662, 2.324	4.628	<.001
£18 vs £15	0.075	−0.069, 0.22	1.344	.179	−0.556	−1.437, 0.325	1.625	.104
£23 vs £18	0.114	−0.030, 0.258	2.038	.042	1.197	0.217, 2.176	3.147	.002
£29 vs £23	0.024	−0.12, 0.169	0.436	.663	0.150	−1.018, 1.317	0.330	.741
£38 vs £29	0.094	−0.05, 0.239	1.684	.092	0.703	−0.688, 2.093	1.301	.193
£54 vs £38	−0.047	−0.191, 0.096	0.848	.397	−0.165	−1.661, 1.331	0.284	.777
Estimate					−1.001	−1.496, −0.505	5.203	<.001
Age	0.087	0.060, 0.115	8.128	<.001	−0.004	−0.18,0.172	0.062	.950
Gender	0.045	−0.033, 0.123	1.483	.138	0.354	−0.157, 0.864	1.786	.074
Effect of condition: *F*(7,1054) = 21.15, *p* < .001					*χ* ^2^(7) = 188.25, *p* < .001			
Overall model: *F*(9,1054) = 23.76, *p* < .001, $R_{adj}^{2}=.162$					*χ* ^2^(10) = 191.61, *p* < .001, Pseudo-*R* ^2^ = .268			

Note: Inferred values (estimates) were log-transformed as log_10_(*x* + 1). $R_{adj}^{2}$ is adjusted *R* ^2^. Pseudo-*R* ^2^ values are Nagelkerke’s *R* ^2^. For row names in upper table: d = day(s). For completeness, we have included tests of overall model fit in these and subsequent regressions, although these were not part of our pre-registered analysis plan.

## Footnotes

1 There are various reasons for this; perhaps the simplest is the operation of competition. For example, a lottery that offers unattractive odds will lose punters to one that offers a better chance of winning, but a lottery that offers an expected return greater than the price of the ticket will go bust.

2 A strong form of this heuristic entails the inference that gambles have expected returns equal to the stake; a more general version entails that people infer larger rewards to have lower probability, and vice-versa.

3 As one illustration, a one-year investment earning the Bank of England’s base rate that started in February 2008 would have been earning 5.25% interest p.a. in the first month but only 1.5% p.a. for the last month.

4 Using the nomenclature Sample.Condition.Estimates.Regression to indicate the sample (full, attentive, or attentive-novice), coding of condition (continuous or factor), coding of estimates (raw or log-transformed), and type of regression (conventional vs robust), the 6 cases were: Full.Factor.Raw.Robust: b = −0.026 [−0.054, 0.002], *p* = .015; At-tentive.Factor.Raw.Robust: b = −0.026 [−0.058, 0.007], *p* = .042; Novice.Factor.Raw.Robust: b = −0.003 [−0.008, 0001], *p* = .053; Novice.Factor.Raw.Conventional: b = −0.003 [−0007, 0001], *p* = .054; Novice.Continuous.Raw.Conventional: b = −0.004 [−0.008, 0.001], *p* = .024.

5 We are grateful to Jon Baron for this suggestion.

## References

[1] Alempaki D, Canic E, Mullett TL, Skylark WJ, Starmer C, Stewart N, Tufano F (2019). Re-examining how utility and weighting functions get their
shapes: a quasi-adversarial collaboration providing a new
interpretation. Management Science.

[2] Albert SM, Duffy J (2012). Differences in risk aversion between young and older
adults. Neuroscience and Neuroeconomics.

[3] Brunswik E (1943). Organismic achievement and environmental
probability. Psychological Review.

[4] Chao L-W, Szrek H, Sousa Pereira N, Pauly MV (2009). Time preference and its relationship with age, health, and
survival probability. Judgment and Decision Making.

[5] Dai J, Pachur T, Pleskac TJ, Hertwig R, Hertwig R, Pleskac TJ, Pachur T (2019). Tomorrow never knows: Why and how uncertainty matters in
intertemporal choice. Taming Uncertainty.

[6] Doyle JR (2013). Survey of time preference, delay discounting
models. Judgment and Decision Making.

[7] Du W, Green L, Myerson J (2002). Cross-cultural comparisons of discounting delayed and
probabilistic rewards. The Psychological Record.

[8] Gigerenzer G, Todd PM (1999). Simple heuristics that make us smart.

[9] Green L, Fry AF, Myerson J (1994). Discounting of delayed rewards. Psychological Science.

[10] Green L, Myerson J (1996). Exponential versus hyperbolic discounting of delayed outcomes:
Risk and waiting time. American Zoologist.

[11] Jimura K, Myerson J, Hilgard J, Keighley J, Braver TS, Green L (2011). Domain independence and stability in young and older adults’
discounting of delayed rewards. Behavioural Processes.

[12] Kirby KN, Petry NM, Bickel WK (1999). Heroin addicts have higher discount rates for delayed rewards
than non-drug-using controls. Journal of Experimental Psychology: General.

[13] Krizan Z, Windschitl PD (2007). The influence of outcome desirability on optimism. Psychological Bulletin.

[14] Laming DRJ (1997). The measurement of sensation.

[15] Leuker C, Pachur T, Hertwig R, Pleskac TJ (2018). Exploiting risk-reward structures in decision making under
uncertainty. Cognition.

[16] Leuker C, Pachur T, Hertwig R, Pleskac TJ (2019a). Do people exploit risk-reward structures to simplify information
processing in risky choice?. Journal of the Economic Science Association.

[17] Leuker C, Pachur T, Hertwig R, Pleskac TJ (2019b). Too good to be true? Psychological responses to uncommon options
in risk-reward environments. Journal of Behavioral Decision Making.

[18] Li Y, Baldassi M, Johnson EJ, Weber EU (2013). Complementary cognitive capabilities, economic decision making,
and aging. Psychology and Aging.

[19] Löckenhoff CE, O’Donoghue T, Dunning D (2011). Age differences in temporal discounting: The role of
dispositional affect and anticipated emotions. Psychology and Aging.

[20] Löckenhoff CE, Samanez-Larkin GR (2020). Age differences in intertemporal choice: The role of task type,
outcome characteristics, and covariates. Journals of Gerontology: Psychological Sciences.

[21] Maechler M, Rousseeuw P, Croux C, Todorov V, Ruckstuhl A, Salibian-Barrera M, Verbeke T, Koller M, Conceicao EL, Anna di Palma M (2020). robust-base: Basic Robust Statistics. R package version 0.93–6.

[22] Mahalingam V, Stillwell D, Kosinski M, Rust J, Kogan A (2014). Who can wait for the future? A personality
perspective. Social Psychological and Personality Science.

[23] Matthews WJ (2012). How much do incidental values affect the judgment of
time?. Psychological Science.

[24] Matthews WJ, Gheorghiu AI, Callan MJ (2016). Why dowe overestimate others’ willingness to pay?. Judgment and Decision Making.

[25] Matthews WJ, Stewart N (2009). Psychophysics and the judgment of price: Judging complex objects
on a non-physical dimension elicits sequential effects like those in
perceptual tasks. Judgment and Decision Making.

[26] McKerchar TL, Green L, Myerson J, Pickford TS, Hill JC, Stout SC (2009). A comparison of four models of delay discounting in
humans. Behavioural Processes.

[27] Myerson J, Baumann AA, Green L (2016). Individual differences in delay discounting: Differences are
quantitative with gains, but qualitative with losses. Journal of Behavioral Decision Making.

[28] Myerson J, Green L (1995). Discounting of delayed rewards: models of individual
choice. Journal of the Experimental Analysis of Behavior.

[29] Odum AL (2011). Delay discounting: Trait variable?. Behavioural Processes.

[30] Oshri A, Hallowell E, Liu S, MacKillop J, Galvan A, Kogan SM, Sweet LH (2019). Socioeco-nomic hardship and delayed reward discounting:
Associations with working memory and emotional reactivity. Developmental Cognitive Neuroscience.

[31] Pleskac TJ, Hertwig R (2014). Ecologically rational choice and the structure of the
environment. Journal of Experimental Psychology: General.

[32] Reimers S, Maylor EA, Stewart N, Chater N (2009). Associations between a one-shot delay discounting measure and
age, income, education and real-world impulsive behavior. Personality and Individual Differences.

[33] Richter D, Mata R (2018). Age differences in intertem-poral choice: U-shaped associations
in a probability sample of German Households. Psychology and Aging.

[34] Rigoli F, Dolan R (2019). Better than expected: The influence of option expectations during
decision-making.

[35] Samuelson PA (1937). A note on measurement of utility. Review of Economic Studies.

[36] Savage LJ (1954). The foundations of statistics.

[37] Shamosh NA, DeYoung CG, Green AE, Reis DL, Johnson MR, Conway ARA, Engle RW, Braver TS, Gray JR (2008). Individual differences in delay discounting: Relation to
intelligence, working memory, and anterior prefrontal cortex. Psychological Science.

[38] Skylark WJ (2018). If John is taller than Jake, where is John? Spatial inference
from magnitude comparison. Journal of Experimental Psychology: Learning, Memory, and
Cognition.

[39] Skylark WJ, Prabhu-Naik S (2018). A new test of the risk-reward heuristic. Judgment and Decision Making.

[40] Stewart N, Chater N, Brown GDA (2006). Decision by sampling. Cognitive Psychology.

[41] Stewart N, Chater N, Stott HP, Reimers S (2003). Prospect relativity: How choice options influence decision under
risk. Journal of Experimental Psychology: General.

[42] Stewart N, Reimers S, Harris AJL (2015). On the origin of utility, weighting, and discounting functions:
How they get their shapes and how to change their shapes. Management Science.

[43] Towe SL, Hobkirk AL, Ye DG, Meade CS (2015). Adaptation of the monetary choice questionnaire to accommodate
extreme monetary discounting in cocaine users. Psychology of Addictive Behaviors.

[44] Tversky A, Simonson I (1993). Context-dependent preferences. Management Science.

[45] Whelan R, McHugh LA (2009). Temporal discounting of hypothetical monetary rewards by
adolescents, adults, and older adults. The Psychological Record.

