# Supplementary material for: The delay-reward heuristic: What do people expect in intertemporal choice tasks?
Source: Judgm Decis Mak. Author manuscript; Available in PMC 2020 Oct 19. (PMC7116214)
Supplement: Appendix [file EMS96525-supplement-Appendix.pdf]

Appendix: Details of Participant Sampling and Eligibility Criteria

Sample sizes were somewhat arbitrary and shaped by budgetary considerations: we simply requested and obtained a fixed number of participants from the recruitment platforms (350 each for Studies 1A and 1B; 2200 in total for Studies 2A and 2B) and removed people as per the exclusion criteria described below. For Studies 1A and 1B (run on MTurk), we "approved" all submissions; for Studies 2A and 2B (run on prolific.co), we rejected a handful of submissions from participants who did not complete the task or who indicated an age under 18, and the platform replaced these until 2200 had been approved.

In all studies, the data collection software (Qualtrics) was set to block IP addresses that were already registered as having completed the task (this system may be imperfect), but it allowed participants with the same IP to re-start the survey after exiting early. For each study we therefore excluded rows in the data file where the IP address had occurred earlier in the study or in one of the previous studies in this series or similar experiments. In the case of overlapping timestamps, both instances were excluded. The demographics sections at the ends of all studies asked "Which of the following best describes you?" with response options: "This is the first time I have completed this survey"; "I have previously started the survey, but did not finish it (e.g., the browser crashed, I lost progress and restarted"); "This is not the first time I have completed this survey; I have previously completed it"). We only included participants who chose the first option. Other details were study-specific, as follows.

Study 1A

We requested 350 participants from MTurk. The study was only visible to participants who had previously completed at least 100 "HITS", who had at least a 98% approval rating, and who were based in the United States. The recruitment page on MTurk told people to use a desktop computer and to only participate if English was their first language, but these weren't checked/enforced. A simple consent page invited participants to click "advance" to signal their consent. Each participant was paid 40 cents for taking part. Data were collected in February 2018.

Study 1B

We requested 350 participants from MTurk, this time via the Turkprime platform (www.cloudresearch.com), which blocks suspicious worker IDs/locations. Participants whose "Worker ID" had appeared in Study 1 were blocked from participating; no requirement was put on number/proportion of completed/approved "HITs" in the past, but participants were requested to be from the United States. The consent form included multiple yes/no questions; a "no" to any question meant the participant was redirected to the end of the survey. Because of concern about "bots" and other suspicious activity on the MTurk platform around the time this study was conducted, we ran the IP addresses and recorded Geo-location data through an online tool (https://itaysisso.shinyapps.io/Bots/) intended to flag potentially suspicious responses; we applied this on November 5th 2019. (Although we could have retrospectively applied this screening to the participants in Study 1, we preferred to retain the sample selected using the eligibility requirements we planned at the time of data collection.) Each participant was paid 40 cents for taking part. Data were collected in December 2018.

Studies 2A and 2B

We requested 2200 participants from prolific.co, with the requirement that participants be resident in the UK, over 18, working on a desktop computer, that they had not previously participated in similar studies that we had run on this platform, and that they had a 95% approval rating on prolific.co. Participants had to complete a "captcha" in order to access the task. The survey used a consent form with yes/no options similar to that for Study 1B. The survey software screened out participants from outside the UK or who were on mobile devices, and those who answered "no" to any consent questions (screened-out participants were asked to "return" the job to Prolific). Each participant was paid 40 pence for taking part. Data were collected in March 2020.
